# Increased *pfmdr1* gene copy number and the decline in *pfcrt* and *pfmdr1* resistance alleles in Ghanaian *Plasmodium falciparum* isolates after the change of anti-malarial drug treatment policy

**DOI:** 10.1186/1475-2875-12-377

**Published:** 2013-10-30

**Authors:** Nancy O Duah, Sena A Matrevi, Dziedzom K de Souza, Daniel D Binnah, Mary M Tamakloe, Vera S Opoku, Christiana O Onwona, Charles A Narh, Neils B Quashie, Benjamin Abuaku, Christopher Duplessis, Karl C Kronmann, Kwadwo A Koram

**Affiliations:** 1 Epidemiology Department, Noguchi Memorial Institute for Medical Research, College of Health Sciences, University of Ghana, PO Box LG581, Legon, Ghana; 2 Centre for Tropical Clinical Pharmacology and Therapeutics, University of Ghana Medical School, PO Box GP4236, Accra, Ghana; 3 United States Naval Medical Research Unit No.3, Cairo, Egypt

**Keywords:** Anti-malarial drug resistance, *Plasmodium falciparum* chloroquine resistance transporter gene (*pfcrt*), *Plasmodium falciparum* multidrug resistance gene (*pfmdr*1), Molecular markers, Ghana

## Abstract

**Background:**

With the introduction of artemisinin-based combination therapy (ACT) in 2005, monitoring of anti-malarial drug efficacy, which includes the use of molecular tools to detect known genetic markers of parasite resistance, is important for first-hand information on the changes in parasite susceptibility to drugs in Ghana. This study investigated the *Plasmodium falciparum* multidrug resistance gene (*pfmdr1*) copy number, mutations and the chloroquine resistance transporter gene (*pfcrt*) mutations in Ghanaian isolates collected in seven years to detect the trends in prevalence of mutations.

**Methods:**

Archived filter paper blood blots collected from children aged below five years with uncomplicated malaria in 2003–2010 at sentinel sites were used. Using quantitative real-time polymerase chain reaction (qRT-PCR), 756 samples were assessed for *pfmdr1* gene copy number. PCR and restriction fragment length polymorphism (RFLP) were used to detect alleles of *pfmdr1* 86 in 1,102 samples, *pfmdr1* 184, 1034, 1042 and 1246 in 832 samples and *pfcrt* 76 in 1,063 samples. Merozoite surface protein 2 (*msp2*) genotyping was done to select monoclonal infections for copy number analysis.

**Results:**

The percentage of isolates with increased *pfmdr1* copy number were 4, 27, 9, and 18% for 2003–04, 2005–06, 2007–08 and 2010, respectively. Significant increasing trends for prevalence of *pfmdr1* N86 (×^2^ = 96.31, p <0.001) and *pfcrt* K76 (×^2^ = 64.50, p <0.001) and decreasing trends in *pfmdr1* Y86 (×^2^ = 38.52, p <0.001) and *pfcrt* T76 (×^2^ = 43.49, p <0.001) were observed from 2003–2010. The *pfmdr1* F184 and Y184 prevalence showed an increasing and decreasing trends respectively but were not significant (×^2^ = 7.39,p=0.060; ×^2^ = 7.49, p = 0.057 respectively). The *pfmdr1* N86-F184-D1246 haplotype, which is alleged to be selected by artemether-lumefantrine showed a significant increasing trend (×^2^ = 20.75, p < 0.001).

**Conclusion:**

Increased *pfmdr1* gene copy number was observed in the isolates analysed and this finding has implications for the use of ACT in the country although no resistance has been reported. The decreasing trend in the prevalence of chloroquine resistance markers after change of treatment policy presents the possibility for future introduction of chloroquine as prophylaxis for malaria risk groups such as children and pregnant women in Ghana.

## Background

Malaria is a childhood killer disease in sub-Saharan Africa. Consequently, the absence of an effective malaria vaccine and low uptake of currently available preventive tools makes chemotherapy a strong pillar of the strategies to control malaria in disease-endemic areas of the world. However, the emergence and spread of clones of *Plasmodium falciparum* which are resistant to most available anti-malarial drugs makes the control of the disease difficult to achieve. Since the first report of chloroquine-resistant *P. falciparum* in Thailand [1] and the rapid spread of resistance worldwide, followed by the recommendation by WHO to use artemisinin-based combination therapy (ACT), many countries in malaria-endemic areas have been monitoring anti-malarial drug resistance. Active implementation of malaria surveillance and the exploitation of molecular as well as phenotypic methods to characterize parasite drug susceptibility profiles will expedite the clarification of spatio-temporal drug sensitivity. This will enable early detection of resistance to the artemisinin derivatives and more efficient use of other available anti-malarial drugs.

Chloroquine resistance has been linked to single nucleotide polymorphisms (SNPs) in the *P. falciparum* transporter gene (*pfcrt*) on chromosome 7 [2]. The level of parasite resistance to this drug is under multilocus/multigenic control [3]. Sets of SNPs in *pfcrt* codons 72, 74, 75, 76, 97, 152, 163, 220, 271, 326, 356 and 371 were associated with chloroquine resistance in *P. falciparum* from Southeast Asia, Africa and South America [4-6]. The mutation on codon 76, that results from the substitution of threonine for lysine in the gene sequence is the seminal SNP for producing the resistance phenotype and the most reliable molecular marker of chloroquine resistance among the various mutations identified [5,6]. In addition there is reported link between T76 and AQ resistance, however the association is comparatively weaker to that of the other aminoquinoline chloroquine [7-11]. Another genetic mechanism of chloroquine resistance is the SNPs in the *P. falciparum* multidrug-resistance gene (*pfmdr*1) on chromosome 5 which encodes a P-glycoprotein homologue-1 multi-drug resistant transporter located in the parasite food vacuole, and is associated with enhanced efflux of the drug from resistant parasites [12]. The *pfmdr1* mutations linked to anti-malarial drug resistance occur at codons 86, 184, 1034, 1042 and 1246 [12-15]. However, the mutation that occurs as a result of the substitution of asparagine for tyrosine at position 86 is linked with chloroquine resistance [12,14,16-19]. Additionally, multiple *pfmdr1* SNPs have been associated with susceptibility profiles of many anti-malarial drugs. These SNPs, as well as the gene amplification, may alter substrate specificity for aminoquinolines and arylaminoalcohols [20] promoting *P. falciparum* resistance to diverse anti-malarials, including chloroquine, mefloquine, quinine and artemisinin derivatives. The Y184F, N1042D and D1246Y mutations are associated with the chloroquine resistance phenotype from samples in Africa, Asia and South America [3,21,22]. The *pfmdr1* haplotype of N86, F184 and D1246 (NFD) were selected in recrudescence samples after artemether-lumefantrine (AL) treatment suggesting that this haplotype conferred a fitness advantage upon AL pressure [23]. With the introduction of AL use in Tanzania and Mozambique, the trends in NFD prevalence have been shown to be increasing [24-26] which is supports the fact of AL pressure. In addition, the *pfmdr1* N86 and *pfcrt* K76 wild-type alleles are also selected by AL treatment but this observation was not made in the use of artesunate-amodiaquine (AS-AQ) and amodiaquine-sulphadoxine-pyrimethamine (AQ-SP) [27]. The *pfmdr1* D1246Y and N86Y mutations predict resistance and recrudescence to AQ and quinine in Uganda [28]. The N86Y mutation increases sensitivity to artemisinin and dihydroartemisinin-piperaquine (DHAP) [20]. The N86Y, Y184F and N1042D mutations increased susceptibility to aryl-amino-alcohol drugs, including mefloquine, halofantrine, lumefantrine, and artemisinin derivatives [28-32]. In South America, the S1034C, N1042D and D1246Y mutations are associated with quinine resistance [33] and are also implicated in increased sensitivity to artemisinin [34,35]. The S1034C and N1042D mutations were reported to reduce or abolish resistance to mefloquine [30].

The *pfmdr1* gene expression levels have been considered in the etiology of the parasite resistance to some anti-malarial drugs and it is being explored in epidemiological studies. Increase in *pfmdr1* gene copy number has been linked to *P. falciparum* diminished susceptibility to anti-malarial drugs, such as mefloquine, AS-MQ and AL combinations [36-38]. Although there is no reported correlation of *pfmdr1* gene copy number and treatment failure, this marker is important for the prediction of recrudescence with the use of the anti-malarials mentioned above [39]. There is an assertion that *pfmdr1* gene copy number rather than the SNPs exercises greater influence in mediating anti-malarial drug resistance to some compounds [40]. This was reported to be due to the fact that many transporter proteins mandate concerted complementary attention to copy number variations (CNV) in mediating anti-malarial activity [40].

Ghana is a malaria-endemic country using ACT (AS-AQ, AL and DHAP) for the treatment of uncomplicated malaria since 2005. Information from molecular investigations, in addition to *in vivo* and *in vitro* analysis, is crucial for early detection and prediction of resistance to the ACT. Before the change of the drug policy in Ghana, the prevalence of the *pfcrt* T76 mutation and the *pfmdr1* Y86 mutation associated with chloroquine resistance ranged from 46 to 98% and 42 to 95%, respectively from five sentinel sites [16]. Due to the Malawian and Kenyan experiences where there was significant decrease in the resistant parasite populations when the drug was withdrawn from use [41,42], it is expected that a similar phenomenon may occur in Ghana. Thus the continuous monitoring of the prevalence of these mutations in parasites in the country is important. Such information is crucial for consideration of chloroquine as prophylaxis for malaria risk groups like children and pregnant women. With the use of ACT in the country, *pfmdr1* copy number determination will help in the early detection of artemisinin derivatives resistance which has implications for chemoprophylactic drug recommendations for natives and travelers from non-endemic regions. This study investigated the prevalence of mutations in *pfcrt* and *pfmdr1* genes as well as the gene copy number of *pfmdr1* in clinical isolates collected from nine sentinel sites in Ghana to detect the trends in the prevalence over seven years.

## Methods

### Study sites

Samples used in this investigation were collected from 2003–2010 from nine sentinel sites in Ghana established for monitoring anti-malarial drug resistance in the country. These sites are located in the three ecological zones of Ghana: Begoro, Bekwai, Hohoe, Sunyani and Tarkwa are located in the tropical forest ecological zone and experience perennial malaria transmission. Navrongo, Wa and Yendi are in the Guinea Savanna ecological zone and experience seasonal malaria transmission. Cape-Coast is in the Coastal Savanna ecological zone and experience perennial malaria transmission.

### Study samples

Blood samples were collected from children aged six to 59 months presenting with uncomplicated malaria at the sentinel sites after the parents or guardians of these children gave informed consent for the children to participate in the study [43-45]. Filter-paper blood blots were made for each child, air-dried and stored in zip-locked bags with silica gel at room temperature until use.

### Determination of *pfmdr1* gene copy number

In all, 756 filter paper, blood-blot samples collected from 2003–2010 were used for the *pfmdr*1 copy number (CN) determination. The real-time quantitative PCR (qRT-PCR) method was used following a published protocol [36]. Parasite DNA was extracted using the QIAamp DNA Blood Mini Kit. The *pfmdr1* gene copy number was estimated for all the parasite isolates by the relative ΔΔCt method [36]. The DNA from *P. falciparum* cell lines 3D7, K1, W2mef and DD2 with copy numbers of 1, 1, 2 and 3–4, respectively were used as references. Samples were run in triplicates for the quantitative PCR and repeated additional times for samples with estimated CN values above 1.5. The estimated CN values were rounded to the nearest integer. For the selection of monoclonal infections from the entire data, the merozoite surface protein 2 (*msp2*) genotyping analysis was done on all 756 samples for FC27 and IC1 genotypes following published protocols [46,47]. Monoclonal infections were selected samples with only one *msp2* genotype; either FC27 or IC1with one PCR band seen on the electrophoresis gel, which were scored by two people.

### Detection of *pfmdr1* and *pfcrt* polymorphisms

In all, 1,102 samples were analysed for the *pfmdr1* polymorphisms at codon 86 (asparagine to tyrosine) and 832 samples for codons 184 (tyrosine to phenylalanine), 1034 (serine to cysteine), 1042 (asparagine to aspartic acid) and 1246 (aspartic acid to tyrosine). For *pfcrt* polymorphism at codon 76 (lysine to threonine), 1,063 samples were analysed. DNA was extracted from the filter-paper blood blots using the Tris EDTA buffer extraction method [48]. PCR followed by restriction fragment length polymorphism (RFLP) was used to detect the mutations using published protocols for *pfmdr1*[13] and *pfcrt*[5].

### Data analysis

The percentage of isolates with increased *pfmdr1* gene copy number was determined for each sentinel site for the time points; 2003–04, 2005–06, 2007–08, and 2010. The data were then pooled for the percentage of Ghanaian isolates with increased copy number and the observed trend determined. The prevalence of mutations for *pfmdr1* gene codons 86, 184, 1034, 1042 and 1246 and *pfcrt* codon 76 were determined for each site and an overall data was pooled from the sites for the Ghanaian isolates analysis. The significance of the observed trends in the prevalence of the alleles over the years from 2003 to 2010 was determined using the Chi-squared test for trends (EpiCalc 2000).

### Ethics

Ethical approval for this study was received from the Noguchi Memorial Institute for Medical Research Institutional Review Board (NMIMR IRB) and the United States Naval Medical Research Unit No 3 (NAMRU-3) IRB.

## Results

### Increased *pfmdr1* gene copy number in Ghanaian isolates

Seven-hundred and fifty-six (756) samples were analysed for the detection of *pfmdr1* gene copy number. Of these, 47 samples (6.3%) were collected in 2003–2004, 171 (22.7%) were collected in 2005–06, 415 (54.9%) collected in 2007–08, and 121 (16%) collected in 2010. From the MSP2 genotyping analysis done, 53.2% (25/47) of the samples from 2003–04 had either FC27 allele (one PCR band seen) or IC1 allele (one PCR band seen) and, therefore, were termed as monoclonal infections. For the 2005–06, 2007–08 and 2010, 48% (82/171), 63.4% (263/415) and 62.8% (76/121), respectively, had monoclonal infections. The total number of samples with monoclonal infections (as defined with MSP2 genotyping) used in the data analysis from 2003–2010 was 446 (59%) out of the 756 samples analysed.

**Figure 1:**
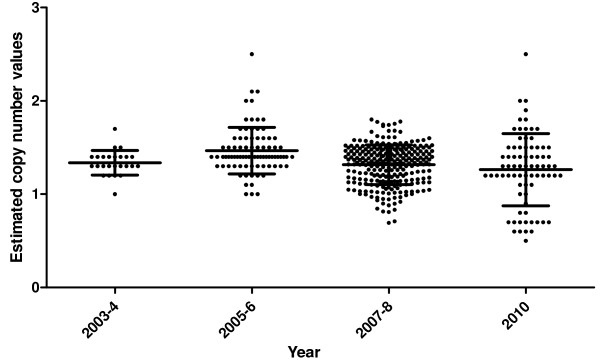
**Estimated copy number values for parasite isolates from Ghana per time points.** Each dot represents the estimated copy number value per parasite. Lines represent the mean and standard deviation of the estimated copy number value for the isolates.

The number of experimental repeats, range and mean ± standard deviation of the estimated copy number (CN) for the *P. falciparum* clones 3D7, K1, W2-mef and Dd2 are shown in Table 1. The samples with estimated CN values above 1.5 were repeated for confirmation. The number of isolates with increased *pfmdr*1 gene copy number from the nine sites per year is shown in Table 2. The sentinel sites with the majority of increased gene copy number per year were Navrongo (4%, 1/25) for 2003–04, Begoro (10%, 8/82) for 2005–06, Yendi (2.3%, 6/263) for 2007–8, and for 2010, Tarkwa, Navrongo and Wa (each with 4%, 3/76). Estimated CN of isolates from monoclonal infections for all the sites per year are shown in Figure 1. The highest estimated CN was 2.5 and the lowest was 0.6 for the isolates with monoclonal infections.

**Table 1:** **Estimated *pfmdr1 *** **gene copy number of laboratory strains used as controls**

	***pfmdr1 *** **CN**	**No. of RT-PCR runs**	**Range of estimated CN**	**Mean estimated CN ± SD**
3D7	1	30	0.76 - 1.40	1.11 ± 0.15
K1	1	30	0.79 - 1.41	1.16 ± 0.16
W2mef	2	4	1.86 - 2.34	2.11 ± 0.22
DD2	3-4	4	3.43 - 4.06	3.77 ± 0.34

**Table 2:** **Percentage of isolates with increased *pfmdr1 *** **gene copy number in monoclonal infections for each site per year**

**Sites**	**%CN> 1**			
	**2003-04**	**2005-06**	**2007-08**	**2010**
Begoro	-	42 (8/19)	0 (0/38)	0 (0/9)
Bekwai	-	0 (0/5)	10 (2/22)	20 (2/10)
Cape-Coast	-	55 (6/11)	3 (1/32)	7 (1/14)
Hohoe	0 (0/12)	-	7 (2/29)	-
Navrongo	8 (1/13)	20 (3/15)	3 (1/29)	30 (3/10)
Sunyani	-	12 (1/8)	10 (3/30)	0 (0/10)
Tarkwa	-	0 (0/2)	27 (4/15)	100 (3/3)
Wa	-	0 (0/2)	12 (4/33)	33 (3/9)
Yendi	-	20 (4/20)	17 (6/35)	18 (2/11)
**Total**	**4 (1/25)**	**27 (22/82)**	**9 (23/263)**	**18 (14/76)**

### Decline in the prevalence of *pfcrt* T76 and *pfmdr1* Y86 alleles after change in drug policy

**Figure 2:**
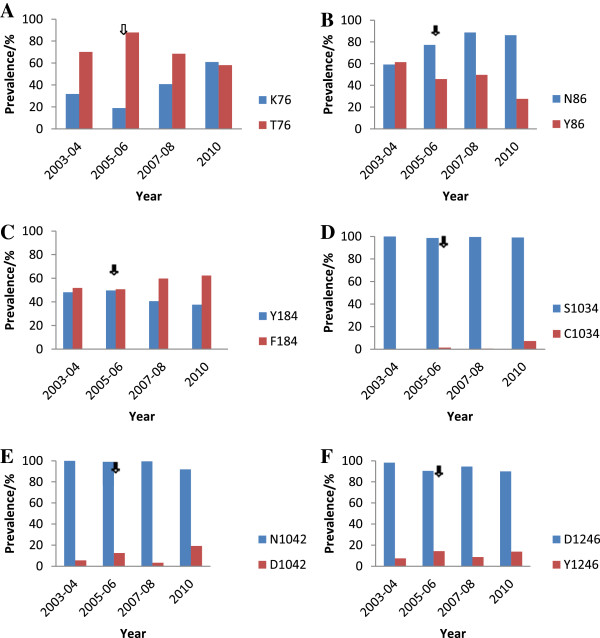
**Trends in the prevalence of *pfcrt *codon 76 and *pfmdr1 *codons 86, 184, 1034, 1042 and 1246 alleles prior to and after the change in treatment policy.** The arrow indicates the period of anti-malarial drug policy change. **(A)***pfcrt* 76, **(B)***pfmdr1* 86, **(C)***pfmdr1* 184, **(D)***pfmdr1* 1034, **(E)***pfmdr1* 1042 and **(F)***pfmdr1* 1246.

In all, 1,063 samples were analysed for the detection of the *pfcrt* codon 76 alleles (K or T) using PCR followed by RFLP. Of this number of isolates, 324 (30.5%) were collected in 2003–04, 232 (21.8%) collected in 2005–06, 402 (37.8%) collected in 2007–08, and 105 (9.9%) were collected in 2010 from the sentinel sites. The prevalence of the T76 (chloroquine resistance allele) as observed from the sentinel sites ranged from 50-98% in 2003–04, 73-95% in 2005–06, 50-95% in 2007–08 and 45-80% in 2010. Whilst for K76 (sensitive allele), the ranges of the prevalence from the sites were 4-52% in 2003–04, 5-30% in 2005–06, 6-62% in 2007, and 25-76% in 2010. With regard to the isolates with both alleles of *pfcrt* codon 76 (K76T), the observed prevalence when the data were pooled for Ghana are 2, 10% 12, and 20% from 2003–04, 2005–06, 2007–07 and 2010, respectively. The observed trend of the prevalence of the alleles over the years before and after the change in drug policy is shown in Figure 2A. The prevalence of the *pfcrt* 76 alleles for all the sites is shown in Additional file 1. A significant decreasing trend in T76 (×^2^ = 43.49, p < 0.001) and an increasing trend in K76 (×^2^ = 64.50, p < 0.001) were observed from 2003 to 2010 (Figure 2A).

The *pfmdr1* codon 86 alleles (N or Y) were detected in 1,102 samples. Of this, 324 (29.4%) samples were collected in 2003–04, 272 (24.6%) were collected in 2005–06, 397 (36%) collected in 2007–08, and 109 (9.9%) in 2010 from the sentinel sites. The prevalence of the resistant allele Y86 for all the individual sites ranged from 48-96% in 2003–04, 31-67% in 2005–06, 36-67% in 2007–08, and 10- 50% in 2010. For the chloroquine-sensitive allele N86, the prevalence ranged from 34-71% in 2003–04, 65-89% in 2005–06, 71-100% in 2007–08, and 80-100% in 2010. A general trend of increase in the prevalence was observed for N86 (×^2^ = 96.31, p < 0.001) and a decrease in Y86 (×^2^ = 38.52, p < 0.001) alleles from 2003 to 2010 (Figure 2B).

### Prevalence of isolates with *pfmdr1* codons 184, 1034, 1042 and 1246 alleles

*Pfmdr1* codons 184 (Y and F), 1034 (S and C), 1042 (D and N) and 1246 (D and Y) alleles were detected in 832 samples. Out of this total number, 54 (6.5%) samples were collected in 2003–04, 272 (32.7%) were collected in 2005–06, 397 (47.7%) were collected in 2007–08, and 109 (13.1%) collected in 2010. The ranges of prevalence of the *pfmdr1* Y184 for all the sites per year are 22-74% for 2003–04, 18-65% for 2005–06, 30-53% for 2007–08, and 20-60% for 2010. For the *pfmdr1* F184 mutant allele, the ranges were 26-78%, 35-82%, 48-70%, and 40-80% for 2003–04, 2005–06, 2007–08, and 2010, respectively. The prevalence of the *pfmdr1* codon 184 alleles for all the sites per year is shown Additional file 1. The observed increasing trend of F184 and the decreasing trend in the prevalence of Y184 alleles did not reach statistical significance (×^2^ = 7.39, p = 0.060; ×^2^ = 7.49, p = 0.057, respectively) (Figure 2C).

The wild type allele of *pfmdr1* 1034 (S1034) was predominant in the isolates from all the time points. The prevalence of the isolates with the allele was 100% for 2003–04, ranged from 92-100%, 98-100%, and 95-100% for 2005–06, 2007–08 and 2010, respectively. The highest prevalence of the mutant C1034 was 35% and was seen in isolates from Wa (2010). The prevalence of the *pfmdr1* codon 1034 alleles for all the sites per year is shown in Additional file 1. The prevalence of the alleles was stable through the time points (Figure 2D).

The *pfmdr1* N1042 allele, which is the wild type allele, was present in majority of the isolates such that all the samples from 2003–04 had the allele (100%). The prevalence of the allele for 2005–06, 2007–08 and 2010 ranged from 97-100%, 98-100% and 65-100%, respectively. For the mutant allele, D1042, the highest prevalence of 73% was observed in isolates from Begoro (2010). The prevalence of the *pfmdr1* codon 1042 alleles for all the sites per year is shown in Additional file 1. There was stability in the prevalence of the *pfmdr1* codon 1042 alleles over the years (Figure 2E).

The codon 1246 wild type allele was present as the majority allele in the isolates for all the time points. The prevalence ranged for 2003–04, 2005–06, 2007–08 and 2010 from 96-100%, 80-100%, 94-100%, and 75-100%, respectively. For the mutant allele, Y1246, the highest prevalence was 35%, which was observed in the isolates from Cape-Coast (2010). The prevalence of the *pfmdr1* codon 1246 alleles for all the sites per year is shown in Additional file 1. The prevalence of the alleles was stable over the years (Figure 2F).

**Figure 3:**
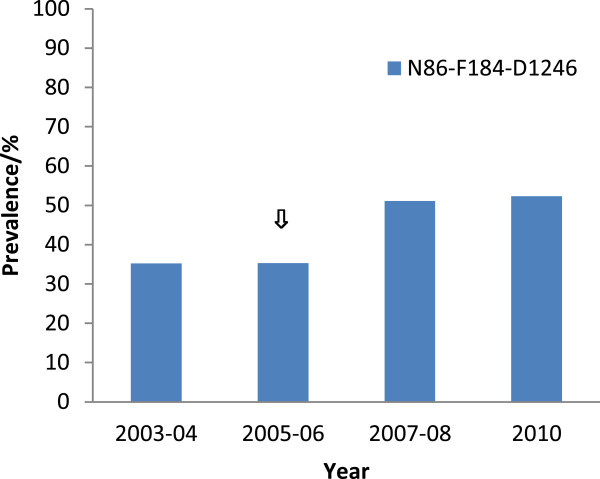
**Trends in the prevalence of *pfmdr1 *N86-F184-D1246 haplotype prior to and after the change in treatment policy.** The arrow indicates the period of anti-malarial drug policy change.

The prevalence of isolates with the *pfmdr1* N86-F184-D1246 (NFD) haplotype showed a significant increasing trend (×^2^ = 20.75, p <0.001) (Figure 3). For the Y86-F184-D1246 haplotype, the prevalence observed for 2003–04, 2005–06, 2007–08, and 2010 was 31.5, 25.0, 31.0 and 18.3%, respectively. Whilst for the Y86-Y184-Y1246 haplotype, the prevalence was 4.0, 6.0, 5.0, and 5.0% for 2003–04, 2005–06, 2007–08, and 2010, respectively. The *pfmdr1* wild type N86-Y184-S1034-N1042-D1246 haplotype was present in 35.2, 33.5, 32.5, and 26.6% of the isolates, respectively for 2003–04, 2005–06, 2007–08, and 2010.

## Discussion

In the absence of any reported parasite resistance to ACT in Ghana, the increased *pfmdr1* gene copy number observed in this study indicates the possibility of nascent clones of *P. falciparum* with reduced susceptibility to the artemisinin derivatives in the country. The high frequency of malaria in Ghana, coupled with a solitary reliance on ACT treatment, poses a situation where selection and propagation of drug-resistant lineages is highly possible. The malaria treatment policy was changed in 2005 from the use of chloroquine as first-line drug to the use of AS-AQ, and in 2008, AL and DHAP were added as the first-line drugs for the treatment of uncomplicated malaria in Ghana. The data showed that before the change, 2003–04, only one parasite out of the 25 isolates tested had increased copy number of 2. This outcome, which is expected, reflects the absence of the artemisinin derivatives and mefloquine drug pressure as these drugs were in limited use at that time. However, it is interesting to note that soon after the implementation of the new malaria drug policy in 2005–06, 27% of the isolates showed an increased *pfmdr1* gene copy number, which was the highest of the four time points. This observation can be attributed to the drug pressure induced by the increased use of the artesunate, a component in the ACT.

Although there is no evidence data on the use of artesunate in the country before 2005, due to the low sensitivity of parasites to chloroquine then, there was limited use of the drug as monotherapy for the treatment of uncomplicated malaria (Neils B. Quashie, pers comm). After 2005–06 there was a decline from 27% in 2005–6 to 9% in 2007–08 with the introduction of the other two ACT, AL and DHAP. There was an increase from 9% to 18% of isolates with copy number increase in 2010. The observed trend of the parasites with increased copy number at the designated time points in the surveillance is suggestive that there is circulation of parasites with reduced sensitivity to artemisinin derivatives and mefloquine in Ghana. Of the three ecological zones in Ghana, isolates from the three savanna zone areas had the majority with increased gene copy number; Navrongo for 2003–04, Yendi for 2007–08, Wa and Navrongo for 2010. It is, therefore, not surprising that Abuaku and others reported a delayed clearance of parasites with the use of AL, especially with parasites from the savannah zone [43]. This finding adds credence to the assertion that gene copy number variation (CNV) may be the suitable indicator for incipient delays in parasite clearance with the use of ACT. CNV in this case involves the duplication of multi-drug resistance genes which help the parasite to survive under unfavourable drug conditions and as such the *pfmdr1* increased copy number is alleged to confer resistance to some ACT and a decrease in the gene copy numbers may confer sensitivity to some anti-malarials [49-51].

The increased *pfmdr1* gene copy number being recorded in sub-Saharan Africa can be worrying although it is not a good indicator of ACT sensitivity in SE Asia it is important to monitor the prevalence of this marker in the circulating parasite population. With ACT use and vector control, the expectation of reduction in mortality and morbidity in African children will be dwindled with the emergence and spread of resistant parasites to the newly introduced ACT. Since the first report of ACT resistance in Southeast Asia and the associated *pfmdr1* gene copy number, the gradual movement of these parasites into Africa is being seen. Increased *pfmdr1* gene copy has been reported in West African countries such as Côte d’Ivoire, Burkina Faso, Togo [52], Senegal [30], but not in Nigeria [23], the Gambia (David J Conway, pers comm), Liberia and Guinea Bissau [53]. Other African countries with reported increased *pfmdr1* copy number in isolates includes Kenya [9], Gabon [31] and Sudan [54] but not in Cameroon [27] and Mozambique [55].

Chloroquine was the first-line drug for the treatment of uncomplicated malaria in Ghana before the change to the use of ACT. In 2007 Koram's group reported the association between the molecular markers of chloroquine resistance, *pfcrt* T76 and *pfmdr1* Y86 with drug treatment failure in a clinical study [16]. This report [16] together with that from *in vivo*[45] and *in vitro*[56] studies ran in parallel, which was made available to the National Malaria Control Programme (NMCP), became one of the basis for treatment policy change in the country. Since then, there has been continuous monitoring of anti-malarial drug resistance in the country using the three aspects of investigation named above. An increasing trend in the prevalence of the *pfcrt* K76 wild type allele from 19 to 59% from 2005 to 2010 and a decreasing trend for *pfcrt* T76 from 88 to 56% for the same period were observed. The general observation depicts a decline of *pfcrt* T76 with and a gradual takeover by K76 after chloroquine was removed in 2005 as the first choice of anti-malarial treatment. This observed trend in Ghana is consistent with observations of decreasing trend of the mutants in Malawi, where the decrease was from 85 to 13% for 1993 to 2000 [41], Kenya, a decrease from 95 to 60% for 1993 to 2006 [42], Senegal, a decrease from 92 to 37% for 2000 to 2010 [30] and Mozambique, from 96 to 32% for 2006 to 2010 [55]. The significant decrease in the prevalence of *pfcrt* T76 in Ghana is indicative of the fitness, which is low for the resistant allele after the selective drug pressure has been withdrawn. This same scenario was observed for *pfmdr1* N86Y, where there was a decline in the prevalence of the mutant from 46 to 28% for 2005 to 2010 and an increase in the prevalence of the wild type strains N86 from 77 to 86%. Similar observations have been made in other studies conducted in Africa where K76T and N86Y were investigated concurrently [30,41,55]. In this study, there was no significant difference between the prevalence of N86 for 2007–08 and 2010, which were 89 and 86%, respectively. This is indicative of a gradual gaining of stability of these genotypes in the population similar to what was observed by Nzila’s group in Kenya [42].

Despite the comparatively faster decline of T76 in the population, the Y86 seems to reduce slowly in this investigation. A study conducted in Gabon with isolates collected from 2004 and 2009, showed stability in T76 with 94% in 2004 and 96% in 2009 whilst the Y86 increased from 16 to 31% for 2004 and 2009 [57]. The apparent disappearance of the chloroquine-resistant parasites with *pfcrt* T76 and *pfmdr1* Y86 from most of the sub-Saharan Africa malarious areas will help in the re-introduction of the cheapest anti-malarial in combination with an artemisinin derivative. The presence of the mutant parasites with *pfcrt* T76 and *pfmdr1* Y86 is a threat to some of the ACT in use. It is important to note that the prevalence of T76 in 2003–04 was 70% and in the year of implementation of AS-AQ in 2005, the prevalence went up to 88%, which is suggestive that another aminoquinoline, in this case, AQ selected the *pfcrt* codon 76 mutants. This is similar to observations made in studies conducted in Uganda and Burkina Faso [11,28,32]. A report from an *in vitro* study conducted in Nigeria showed an association between the T76 mutation and decreased susceptibility to artemether [58]. The increasing trend in the prevalence of N86 observed in this study must be closely monitored since this allele is reportedly linked to decreased sensitivity of parasites to lumefantrine and mefloquine and it is also a marker for lumefantrine tolerance [57]. In addition an increasing trend for K76 will create a future problem for ACT use because it has been seen in recrudescent samples after AL use [59].

The prevalence of the other SNPs of the *pfmdr1* gene was also investigated, which included the alleles of codons 184, 1034, 1042 and 1246. The mutant allele of codon 184 is known to be selected after AL use in recrudescent samples [54,60]. From the results, in 2003–04, the prevalence was about 52% and it reduced to 51% in 2005 and then increased to 62% in 2010. Although the increasing trend of the prevalence of F184 from 2003 to 2010 was not significant, there is an indication of selection of this suggested AL resistance allele. Results from a 2010 study conducted in seven communities in southern Ghana revealed the prevalence of F184 to be about 72% [61]. In the West African region, a report from a study conducted in Senegal also showed the prevalence of 68% in samples collected in 2009–2010 [30]. The wild type allele, Y184 was observed to be decreasing in prevalence whilst the other alleles, such as the S1034 wild type allele, were stable over the time points. An increase in the prevalence of the mutant (C1034) was observed for 2010. For the codon 1042 alleles, the wild type N1042 was also stable until a decrease in prevalence (from 100 to 92%) was observed in 2010. The mutant D1042 was also about 19% in 2010 with low to non-existent levels in the previous years. For the codon 1246 alleles, D1246 dropped from 98% in 2003–04 to 90% in 2010 whilst the Y1246 was observed to be gradually increasing 7% in 2003–04 to 14% in 2010. The Y1246 mutant has been linked to quinine failure [57], as such the use of quinine as second-line drug in Ghana for the treatment of uncomplicated malaria may delay the selection of these mutant parasites. There is more stability in the three *pfmdr1* codons 1034, 1042 and 1246 compared to the 86 and 184. The haplotype mutant of *pfmdr1* C1034-D1042-Y1246 has been linked to increased sensitivity to artemisinin [34,35] whilst C1034-D1042 is linked to sensitivity to mefloquine [30]. Furthermore, the Y86-F184-D1042 mutations have been shown to increase the susceptibility of parasites to aryl-amino-alcohol drugs, artemisinin derivatives, mefloquine, halofantrine and lumefantrine [28-32]. As such the stability of these mutants as observed in this study will help maintain the use of ACT in the country. The N86-F184-D1246 haplotype, which was detected in 35% of the isolates in 2003–04, increased significantly to 53% in 2010. Similar trends in prevalence for the NFD haplotype have been reported in Tanzania and Mozambique after the introduction of AL use [24-26]. In addition, this haplotype as well as *pfcrt* K76 were seen in recrudescent samples after AL use [54,59]. The NFD haplotype therefore needs to be monitored effectively especially in malarious countries using AL for chemotherapy.

## Conclusions

Generally, the observations made in this study with the prevalence of the molecular markers are in line with what is expected before and after the change of the malaria treatment policy. The increase in *pfmdr1* gene copy number (although not a good indicator) in some of the isolates, which is linked to susceptibility of artemisinin derivatives, mefloquine, halofantrine and lumefantrine, has implications for ACT use in Ghana. As the chloroquine resistant genotypes decrease in frequency, and subsequently *in-vitro* data confirms increased susceptibility to the drug, it can be introduced as prophylaxis for malaria risk groups, such as children and pregnant women. The findings draw attention to the need to continually monitor molecular markers of all the anti-malarial drugs currently in use in Ghana to allow for early detection of reduced or increased parasite susceptibility to the drugs. It must be stressed that findings from this study have given some insight into the genetic background of the parasites in circulation in Ghana. This will enable genuine conjuncture into the future of malaria in the country in the context of the SNPs of anti-malarial drug resistance and the potential effect of the increasing trends of the drug-resistant parasite populations. Further monitoring of these markers are ongoing in Ghana for ACT efficacy, including both *in vivo* and *in vitro* studies.

## Competing interests

The authors declared that they have no competing interests.

## Authors’ contributions

NOD, KCK, NBQ, BA and KAK conceived and designed the study. NOD, SAM, DKDS, DDB, MMT, VSO, COO and CAN did the molecular characterization of drug-resistant genes, *pfcrt* and *pfmdr1* and acquired the molecular data. NOD and BA did the data analysis. NOD drafted the manuscript. KCK, KAK and CD coordinated the research work. All authors read and approved the final manuscript.

## Supplementary Material

Additional file 1 **Prevalence of *Plasmodium falciparum *** **infections with *pfcrt *** **and *pfmdr1 *** **alleles at the nine sentinel sites in Ghana for the study time points.** Click here for file

## References

[B1] Kain KC Gopinath R Yau Y Temahivong T Wongsrichanalai C In vivo response of falciparum malaria to chloroquine in Southern Thailand J Infect Dis 1994 170 258 259 10.1093/infdis/170.1.258 8014517

[B2] Fidock DA Nomura T Talley AK Mutations in the *Plasmodium falciparum* digestive vacuole transmembrane protein Pfcrt and evidence for their role in chloroquine resistance Mol Cell 2000 6 861 871 10.1016/S1097-2765(05)00077-8 11090624 PMC2944663

[B3] Setthaudom C Tan-ariya P Sitthichot N Khositnithikul R Suwandittakul N Leelayoova S Mungthin M Role of *Plasmodium falciparum* chloroquine resistance transporter and multidrug resistance 1 genes on in vitro chloroquine resistance in isolates of *Plasmodium falciparum* from Thailand Am J Trop Med Hyg 2011 85 606 611 10.4269/ajtmh.2011.11-0108 21976558 PMC3183763

[B4] Ibrahim ML Steenkeste N Khim N Adam HH Konaté L Coppée JY Ariey F Duchemin JB Field-based evidence of fast and global increase of *Plasmodium falciparum* drug-resistance by DNA-microarrays and PCR/RFLP in Niger Malar J 2009 8 32 10.1186/1475-2875-8-32 19236701 PMC2654903

[B5] Djimde A Doumbo OK Cortese JF Kayentao K Doumbo S Diourte Y Dicko A Su X-Z Nomura T Fidock DA Wellems TE Plowe CV A molecular marker for chloroquine-resistant falciparum malaria N Eng J Med 2001 344 257 263 10.1056/NEJM200101253440403 11172152

[B6] Plowe CV Wellems TE Detection of mutations in a putative *Plasmodium falciparum* transporter linked to chloroquine resistance Report for the WHO Workshop on Markers of Antimalarial Drug Resistance 1999

[B7] Danquah I Coulibaly B Meissner P Petruschke I Müller O Mockenhaupt FP Selection of pfmdr1 and pfcrt alleles in amodiaquine treatment failure in north-western Burkina Faso Acta Trop 2010 111 63 66 20060374 10.1016/j.actatropica.2009.12.008

[B8] Dokomajilar C Lankoande ZM Dorsey G Zongo I Ouedraogo JB Rosenthal PJ Roles of specific *Plasmodium falciparum* mutations in resistance to amodiaquine and sulfadoxine-pyrimethamine in Burkina Faso Am J Trop Med Hyg 2006 75 162 165 16837725

[B9] Holmgren G Björkman A Gil JP Amodiaquine resistance is not related to rare findings of pfmdr1 gene amplifications in Kenya Trop Med Int Health 2006 11 1808 1812 10.1111/j.1365-3156.2006.01742.x 17176345

[B10] Ochong EO van den Broek IV Keus K Nzila A Association between chloroquine and amodiaquine resistance and allelic variation in the *Plasmodium falciparum* multiple drug resistance 1 gene and the chloroquine resistance transporter gene in isolates from the upper Nile in southern Sudan Am J Trop Med Hyg 2003 69 184 187 13677373

[B11] Tinto H Guekoun L Zongo I Guiguemdé RT D'Alessandro U Ouédraogo JB Chloroquine-resistance molecular markers (Pfcrt T76 and Pfmdr-1 Y86) and amodiaquine resistance in Burkina Faso Trop Med Int Health 2008 13 238 240 10.1111/j.1365-3156.2007.01995.x 18304270

[B12] Foote SJ Kyle DE Martin RK Oduola AMJ Forsyth K Kemp DJ Cowman AF Several alleles of the multidrug resistance gene are closely linked to chloroquine resistance in *Plasmodium falciparum* Nature 1990 345 255 258 10.1038/345255a0 2185424

[B13] Duraisingh MT Jones P Sambou I von Seidlein L Pinder M Warhurst DC The tyrosine-86 allele of the pfmdr1 gene of *Plasmodium falciparum* is associated with increased sensitivity to the anti-malarials mefloquine and artemisinin Mol Biochem Parasitol 2000 108 13 23 10.1016/S0166-6851(00)00201-2 10802315

[B14] Wellems TE Panton LJ Gluzman IY Do Rosario VE Gwadz RW Walker-Jonah A Krogstad DJ Chloroquine resistance linked to mdr-like genes in a *Plasmodium falciparum* cross Nature 1990 345 253 255 10.1038/345253a0 1970614

[B15] Wellems TE Walker-Jonah A Panton LJ Genetic mapping of the chloroquine-resistance locus on *Plasmodium falciparum* chromosome 7 Proc Natl Acad Sci U S A 1991 88 3382 3386 10.1073/pnas.88.8.3382 1673031 PMC51451

[B16] Duah NO Wilson MD Ghansah A Abuaku B Edoh D Quashie NB Koram KA Mutations in *Plasmodium falciparum* chloroquine resistance transporter and multidrug resistance genes, and treatment outcomes in Ghanaian children with uncomplicated malaria J Trop Pediatr 2007 53 27 31 17158810 10.1093/tropej/fml076

[B17] Duraisingh MT Drakeley CJ Muller O Bailey R Snounou G Targett GA Greenwood BM Warhurst DC Evidence for selection for the tyrosine-86 allele of the pfmdr 1 gene of *Plasmodium falciparum* by chloroquine and amodiaquine Parasitol 1997 114 205 211 10.1017/S0031182096008487 9075340

[B18] Khalil IF Alifrangis M Tarimo DS Staalsø T Satti GM Theander TG Rønn AM Bygbjerg IC The roles of the pfcrt 76 T and pfmdr1 86Y mutations, immunity and the initial level of parasitaemia, in predicting the outcome of chloroquine treatment in two areas with different transmission intensities Ann Trop Med Parasitol 2005 99 441 448 10.1179/136485905X46441 16004703

[B19] Babiker HA Pringle SJ Abdel-Muhsin A Mackinnon M Hunt P Walliker D High-level chloroquine resistance in Sudanese isolates of *Plasmodium falciparum* is associated with mutations in the chloroquine resistance transporter gene pfcrt and the multidrug resistance Gene pfmdr1 J Infect Dis 2001 183 1535 1538 10.1086/320195 11319692

[B20] Pillai DR Lau R Khairnar K Lepore R Via A Staines HM Krishna S Artemether resistance in vitro is linked to mutations in PfATP6 that also interact with mutations in PfMDR1 in travellers returning with *Plasmodium falciparum* infections Malar J 2012 11 131 10.1186/1475-2875-11-131 22540925 PMC3422158

[B21] Ménard D Yapou F Manirakiza A Djalle D Matsika-Claquin MD Talarmin A Polymorphisms in pfcrt, pfmdr1, dhfr genes and in vitro responses to antimalarials in *Plasmodium falciparum* isolates from Bangui, Central African Republic Am J Trop Med Hyg 2006 75 381 387 16968910

[B22] Pickard AL Wongsrichanalai C Purfield A Kamwendo D Emery K Zalewski C Kawamoto F Miller RS Meshnick SR Resistance to antimalarials in Southeast Asia and genetic polymorphisms in pfmdr1 Antimicrob Agents Chemother 2003 47 2418 2423 10.1128/AAC.47.8.2418-2423.2003 12878499 PMC166057

[B23] Happi CT Gbotosho GO Folarin OA Sowunmi A Hudson T O'Neil M Milhous W Wirth DF Oduola AM Selection of *Plasmodium falciparum* multidrug resistance gene 1 alleles in asexual stages and gametocytes by artemether-lumefantrine in Nigerian children with uncomplicated falciparum malaria Antimicrob Agents Chemother 2009 53 888 895 10.1128/AAC.00968-08 19075074 PMC2650543

[B24] Malmberg M Ngasala B Ferreira PE Larsson E Jovel I Hjalmarsson A Petzold M Premji Z Gil JP Bjorkman A Martensson A Temporal trends of molecular markers associated with artemether-lumefantrine tolerance/resistance in Bagamoyo district, Tanzania Malar J 2013 12 103 10.1186/1475-2875-12-103 23506218 PMC3732084

[B25] Thomsen TT Ishengoma DS Mmbando BP Lusingu JP Vestergaard LS Theander TG Lemnge MM Bygbjerg IC Alifrangis M Prevalence of single nucleotide polymorphisms in the *Plasmodium falciparum* multidrug resistance gene (Pfmdr-1) in Korogwe District in Tanzania before and after introduction of artemisinin-based combination therapy Am J Trop Med Hyg 2011 85 979 983 10.4269/ajtmh.2011.11-0071 22144430 PMC3225174

[B26] Thomsen TT Madsen LB Hansson HH Tomas EV Charlwood D Bygbjerg IC Alifrangis M Rapid selection of *Plasmodium falciparum* chloroquine resistance transporter gene and multidrug resistance gene-1 haplotypes associated with past chloroquine and present artemether-lumefantrine use in Inhambane District, southern Mozambique Am J Trop Med Hyg 2013 88 536 541 10.4269/ajtmh.12-0525 23382159 PMC3592537

[B27] Menard S Morlais I Tahar R Sayang C Mayengue PI Iriart X Benoit-Vical F Lemen B Magnaval JF Awono-Ambene P Basco LK Berry A Molecular monitoring of *Plasmodium falciparum* drug susceptibility at the time of the introduction of artemisinin-based combination therapy in Yaoundé Cameroon: implications for the future. Malar J 2012 11 113 10.1186/1475-2875-11-113 PMC3368752 22498364

[B28] Nsobya SL Kiggundu M Nanyunja S Joloba M Greenhouse B Rosenthal PJ In vitro sensitivities of *Plasmodium falciparum* to different antimalarial drugs in Uganda Antimicrob Agents Chemother 2010 54 1200 1206 10.1128/AAC.01412-09 20065051 PMC2825959

[B29] Uhlemann AC McGready R Ashley EA Brockman A Singhasivanon P Krishna S White NJ Nosten F Price RN Intrahost selection of *Plasmodium falciparum* pfmdr1 alleles after antimalarial treatment on the northwestern border of Thailand J Infect Dis 2007 195 134 141 10.1086/509809 17152017 PMC4337981

[B30] Wurtz N Fall B Pascual A Diawara S Sow K Baret E Diatta B Fall KB Mbaye PS Fall F Diémé Y Rogier C Bercion R Briolant S Wade B Pradines B Prevalence of molecular markers of *Plasmodium falciparum* drug resistance in Dakar Senegal. Malar J 2012 11 197 10.1186/1475-2875-11-197 PMC3470961 22694921

[B31] Nkhoma S Nair S Mukaka M Molyneux ME Ward SA Anderson TJ Parasites bearing a single copy of the multi-drug resistance gene (pfmdr-1) with wild-type SNPs predominate amongst *Plasmodium falciparum* isolates from Malawi Acta Trop 2009 111 76 81 10.1016/j.actatropica.2009.01.011 PMC2746100 19426667

[B32] Nawaz F Nsobya SL Kiggundu M Joloba M Rosenthal PJ Selection of parasites with diminished drug susceptibility by amodiaquine-containing antimalarial regimens in Uganda J Infect Dis 2009 200 1650 1657 10.1086/647988 19905933 PMC2782860

[B33] Meng H Zhang R Yang H Fan Q Su X Miao J Cui L Yang Z In vitro sensitivity of *Plasmodium falciparum* clinical isolates from the China-Myanmar border area to quinine and association with polymorphism in the Na+/H + exchanger Antimicrob Agents Chemother 2010 54 4306 4313 10.1128/AAC.00321-10 20643902 PMC2944579

[B34] Chavchich M Gerena L Peters J Chen N Cheng Q Kyle DE Role of pfmdr1 amplification and expression in induction of resistance to artemisinin derivatives in *Plasmodium falciparum* Antimicrob Agents Chemother 2010 54 2455 2464 10.1128/AAC.00947-09 20350946 PMC2876417

[B35] Mungthin M Khositnithikul R Sitthichot N Suwandittakul N Wattanaveeradej V Ward SA Na-Bangchang K Association between the pfmdr1 gene and in vitro artemether and lumefantrine sensitivity in Thai isolates of *Plasmodium falciparum* Am J Trop Med Hyg 2010 83 1005 1009 10.4269/ajtmh.2010.10-0339 21036827 PMC2963959

[B36] Price RN Uhlemann AC Brockman A McGready R Ashley E Phaipun L Patel R Laing K Looareesuwan S White NJ Nosten F Krishna S Mefloquine resistance in *Plasmodium falciparum* and increased pfmdr1 gene copy number Lancet 2004 364 438 447 10.1016/S0140-6736(04)16767-6 15288742 PMC4337987

[B37] Price RN Cassar C Brockman A Duraisingh M van Vugt M White NJ Nosten F Krishna S The pfmdr1 gene is associated with a multidrug-resistant phenotype in *Plasmodium falciparum* from the western border of Thailand Antimicrob Agents Chemother 1999 43 2943 2949 10582887 10.1128/aac.43.12.2943 PMC89592

[B38] Wilson CM Volkman SK Thaithong S Martin RK Kyle DE Milhous WK Wirth DF Amplification of pfmdr 1 associated with mefloquine and halofantrine resistance in *Plasmodium falciparum* from Thailand Mol Biochem Parasitol 1993 57 151 160 10.1016/0166-6851(93)90252-S 8426608

[B39] Na-Bangchang K Muhamad P Ruaengweerayut R Chaijaroenkul W Karbwang J Identification of resistance of *Plasmodium falciparum* to artesunate-mefloquine combination in an area along the Thai-Myanmar border: integration of clinico-parasitological response, systemic drug exposure, and in vitro parasite sensitivity Malar J 2013 12 263 10.1186/1475-2875-12-263 23898808 PMC3737112

[B40] Anderson TJ Nair S Qin H Singlam S Brockman A Paiphun L Nosten F Are transporter genes other than the chloroquine resistance locus (pfcrt) and multidrug resistance gene (pfmdr) associated with antimalarial drug resistance? Antimicrob Agents Chemother 2005 49 2180 2188 10.1128/AAC.49.6.2180-2188.2005 15917511 PMC1140548

[B41] Kublin JG Cortese JF Njunju EM Mukadam RA Wirima JJ Kazembe PN Djimdé AA Kouriba B Taylor TE Plowe CV Reemergence of chloroquine-sensitive *Plasmodium falciparum* malaria after cessation of chloroquine use in Malawi J Infect Dis 2003 187 1870 1875 10.1086/375419 12792863

[B42] Mwai L Ochong E Abdirahman A Kiara SM Ward S Kokwaro G Sasi P Marsh K Borrmann S Mackinnon M Nzila A Chloroquine resistance before and after its withdrawal in Kenya Malar J 2009 8 106 10.1186/1475-2875-8-106 19450282 PMC2694831

[B43] Abuaku B Duah N Quaye L Quashie N Koram K Therapeutic efficacy of artemether-lumefantrine combination in the treatment of uncomplicated malaria among children under five years of age in three ecological zones in Ghana Malar J 2012 11 388 10.1186/1475-2875-11-388 23173737 PMC3519607

[B44] Koram K Quaye L Abuaku B Efficacy of amodiaquine/artesunate combination therapy for uncomplicated malaria in children under five years in Ghana Ghana Med J 2008 42 55 60 19180204 PMC2631265

[B45] Koram KA Abuaku B Duah N Quashie N Comparative efficacy of antimalarial drugs including ACTs in the treatment of uncomplicated malaria among children under 5 years in Ghana Acta Trop 2005 95 194 203 10.1016/j.actatropica.2005.06.018 16054584

[B46] Enosse S Dobaño C Quelhas D Aponte JJ Lievens M Leach A Sacarlal J Greenwood B Milman J Dubovsky F Cohen J Thompson R Ballou WR Alonso PL Conway DJ Sutherland CJ RTS, S/AS02A malaria vaccine does not induce parasite CSP T cell epitope selection and reduces multiplicity of infection PLoS Clin Trials 2006 1 e5 10.1371/journal.pctr.0010005 16871327 PMC1488895

[B47] Snounou G Zhu X Siripoon N Jarra W Thaithong S Brown KN Viriyakosol S Biased distribution of msp1 and msp2 allelic variants in *Plasmodium falciparum* populations in Thailand Trans R Soc Trop Med Hyg 1999 93 369 374 10.1016/S0035-9203(99)90120-7 10674079

[B48] Bereczky S Martensson A Gil JP Farnert A Rapid DNA extraction from archive blood spots on filter paper for genotyping of *Plasmodium falciparum* Am J Trop Med Hyg 2005 72 249 251 15772315

[B49] Price RN Uhlemann AC van Vugt M Brockman A Hutagalung R Nair S Nash D Singhasivanon P Anderson TJ Krishna S White NJ Nosten F Molecular and pharmacological determinants of the therapeutic response to artemether-lumefantrine in multidrug-resistant *Plasmodium falciparum* malaria Clin Infect Dis 2006 42 1570 1577 10.1086/503423 16652314 PMC4337983

[B50] Sidhu AB Uhlemann AC Valderramos SG Valderramos JC Krishna S Fidock DA Decreasing pfmdr1 copy number in *Plasmodium falciparum* malaria heightens susceptibility to mefloquine, lumefantrine, halofantrine, quinine, and artemisinin J Infect Dis 2006 194 528 535 10.1086/507115 16845638 PMC2978021

[B51] Uhlemann AC Krishna S Antimalarial multi-drug resistance in Asia: mechanisms and assessment Curr Top Microbiol Immunol 2005 295 39 53 10.1007/3-540-29088-5_2 16265886

[B52] Witkowski B Iriart X Soh PN Menard S Alvarez M Naneix-Laroche V Marchou B Magnaval JF Benoit-Vical F Berry A Pfmdr1 amplification associated with clinical resistance to mefloquine in West Africa: implications for efficacy of artemisinin combination therapies J Clin Microbiol 2010 48 3797 3799 10.1128/JCM.01057-10 20668121 PMC2953132

[B53] Ursing J Kofoed PE Rombo L Gil JP No pfmdr1 amplifications in samples from Guinea-Bissau and Liberia collected between 1981 and 2004 J Infect Dis 2006 194 716 719 16897676 10.1086/506456

[B54] Gadalla NB Adam I Elzaki SE Bashir S Mukhtar I Oguike M Gadalla A Mansour F Warhurst D El-Sayed BB Sutherland CJ Increased pfmdr1 copy number and sequence polymorphisms in *Plasmodium falciparum* isolates from Sudanese malaria patients treated with artemether-lumefantrine Antimicrob Agents Chemother 2011 55 5408 5411 10.1128/AAC.05102-11 21896916 PMC3195064

[B55] Raman J Mauff K Muianga P Mussa A Maharaj R Barnes KI Five years of antimalarial resistance marker surveillance in Gaza Province, Mozambique, following artemisinin-based combination therapy roll out PLoS One 2011 6 e25992 10.1371/journal.pone.0025992 22022487 PMC3195082

[B56] Quashie NB Duah NO Abuaku B Koram KA The in-vitro susceptibilities of Ghanaian *Plasmodium falciparum* to antimalarial drugs Ann Trop Med Parasitol 2007 101 391 398 10.1179/136485907X176553 17550644

[B57] Lekana-Douki JB Dinzouna Boutamba SD Zatra R Zang Edou SE Ekomy H Bisvigou U Toure-Ndouo FS Increased prevalence of the *Plasmodium falciparum* Pfmdr1 86N genotype among field isolates from Franceville, Gabon after replacement of chloroquine by artemether-lumefantrine and artesunate-mefloquine Infect Genet Evol 2011 11 512 517 10.1016/j.meegid.2011.01.003 21251998

[B58] Bustamante C Folarin OA Gbotosho GO Batista CN Mesquita EA Brindeiro RM Tanuri A Struchiner CJ Sowunmi A Oduola A Wirth DF Zalis MG Happi CT **In vitro-reduced susceptibility to artemether in **** *P* ****. **** *falciparum * ****and its association with polymorphisms on transporter genes.** J Infect Dis 2012 206 324 332 10.1093/infdis/jis359 22615315 PMC3490696

[B59] Sisowath C Strömberg J Mårtensson A Msellem M Obondo C Björkman A Gil JP In vivo selection of *Plasmodium falciparum* pfmdr1 86 N coding alleles by artemether-lumefantrine (Coartem) J Infect Dis 2005 191 1014 1017 10.1086/427997 15717281

[B60] Zeile I Gahutu JB Shyirambere C Steininger C Musemakweri A Sebahungu F Karema C Harms G Eggelte TA Mockenhaupt FP Molecular markers of *Plasmodium falciparum* drug resistance in southern highland Rwanda Acta Trop 2012 121 50 54 10.1016/j.actatropica.2011.09.009 21996622

[B61] Kwansa-Bentum B Ayi I Suzuki T Otchere J Kumagai T Anyan WK Osei JH Asahi H Ofori MF Akao N Wilson MD Boakye DA Ohta N *Plasmodium falciparum* isolates from southern Ghana exhibit polymorphisms in the SERCA-type PfATPase6 though sensitive to artesunate in vitro Malar J 2011 10 187 10.1186/1475-2875-10-187 21745377 PMC3146903

