# Supplementary material for: Increased pfmdr1 gene copy number and the decline in pfcrt and pfmdr1 resistance alleles in Ghanaian Plasmodium falciparum isolates after the change of anti-malarial drug treatment policy
Source: Malar J. 2013 Oct 30;12:377. doi: 10.1186/1475-2875-12-377 (PMC3819684; doi:10.1186/1475-2875-12-377)
Supplement: Additional file 1 — Prevalence of Plasmodium falciparum infections with pfcrt and pfmdr1 alleles at the nine sentinel sites in Ghana for the study time points. [file 1475-2875-12-377-S1.docx]

**Table 3** **Prevalence of *Plasmodium falciparum* infections with *pfcrt* and *pfmdr1* alleles at the nine sentinel sites in Ghana for the study time points**

| **Site/year** | **K76** | **T76** | **N86** | **Y86** | **Y184** | **F184** | **S1034** | **C1034** | **N1042** | **D1042** | **D1246** | **Y1246** |
| --- | --- | --- | --- | --- | --- | --- | --- | --- | --- | --- | --- | --- |
| **Begoro** |  |  |  |  |  |  |  |  |  |  |  |  |
| 2003-04 | - | - | - | - | - | - | - | - | - | - | - | - |
| 2005-06 | 8  (3/39) | 92  (36/39) | 74  (29/39) | 67  (26/39) | 18  (7/39) | 82  (32/39) | 100  (39/39) | 0  (0/39) | 100  (39/39) | 36  (14/39) | 97  (38/39) | 13  (5/39) |
| 2007-08 | 22  (11/50) | 78  (39/50) | 90  (45/50) | 60  (30/50) | 30  (15/50) | 70  (35/50) | 100  (50/50) | 0  (0/50) | 100  (50/50) | 0  (0/50) | 96  (48/50) | 6  (3/50) |
| 2010 | 50  (7/14) | 57  (8/14) | 80  (12/15) | 27  (4/15) | 20  (3/15) | 80  (12/15) | 100  (15/15) | 0  (0/15) | 93  (14/15) | 73  (11/15) | 100  (15/15) | 7  (1/15) |
| **Bekwai** |  |  |  |  |  |  |  |  |  |  |  |  |
| 2003-04 | - | - | - | - | - | - | - | - | - | - | - | - |
| 2005-06 | 35  (14/40) | 70  (28/40) | 65  (26/40) | 45  (18/40) | 65  (26/40) | 35  (14/40) | 100  (40/40) | 0  (0/40) | 100  (40/40) | 23  (9/40) | 80  (32/40) | 23  (9/40) |
| 2007-08 | 42  (21/50) | 64  (32/50) | 88  (42/48) | 48  (23/48) | 31  (15/48) | 69  (33/48) | 100  (48/48) | 0  (0/48) | 100  (48/48) | 0  (0/48) | 100  (48/48) | 6  (3/48) |
| 2010 | 71  (10/14) | 57  (8/14) | 93  (13/14) | 43  (6/14) | 21  (3/14) | 79  (11/14) | 100  (14/14) | 0  (0/14) | 100  (14/14) | 7  (1/14) | 100  (14/14) | 0  (0/14) |
| **Cape-Coast** |  |  |  |  |  |  |  |  |  |  |  |  |
| 2003-04 | - | - | - | - | - | - | - | - | - | - | - | - |
| 2005-06 | 5  (2/39) | 95  (37/39) | 67  (26/39) | 59  (23/39) | 54  (21/39) | 46  (18/39) | 100  (39/39) | 0  (0/39) | 100  (39/39) | 15  (6/39) | 100  (39/39) | 8  (3/39) |
| 2007-08 | 10  (4/42) | 95  (40/42) | 84  (36/43) | 49  (21/43) | 42  (18/43) | 58  (25/43) | 100  (43/43) | 0  (0/43) | 100  (43/43) | 0  (0/43) | 95  (41/43) | 7  (3/43) |
| 2010 | 25  (5/20) | 80  (16/20) | 80  (16/20) | 50  (10/20) | 20  (4/20) | 80  (16/20) | 100  (20/20) | 0  (0/20) | 100  (20/20) | 0  (0/20) | 75  (15/20) | 35  (7/20) |
| **Hohoe** |  |  |  |  |  |  |  |  |  |  |  |  |
| 2003-04 | 26  (20/78) | 77  (60/78) | 64  (50/78) | 64  (50/78) | 22  (6/28) | 78  (22/28) | 100  (28/28) | 0  (0/28) | 100  (28/28) | 11  (3/28) | 96  (26/28) | 11  (3/28) |
| 2005-06 | - | - | - | - | - | - | - | - | - | - | - | - |
| 2007-08 | 54  (27/50) | 56  (28/50) | 71  (35/49) | 59  (29/49) | 49  (24/49) | 51  (25/49) | 100  (49/49) | 0  (0/49) | 98  (48/49) | 2  (1/49) | 96  (47/49) | 4  (2/49) |
| 2010 | - | - | - | - | - | - | - | - | - | - | - | - |
| **Navrongo** |  |  |  |  |  |  |  |  |  |  |  |  |
| 2003-04 | 26  (9/34) | 74  (25/34) | 71  (24/35) | 50  (17/35) | 74  (20/27) | 26  (7/27) | 100  (27/27) | 0  (0/27) | 100  (27/27) | 0  (0/27) | 100  (27/27) | 4  (1/27) |
| 2005-06 | 30  (12/40) | 88  (35/40) | 85  (34/40) | 38  (15/40) | 58  (23/40) | 43  (17/40) | 100  (40/40) | 0  (0/40) | 100  (40/40) | 3  (1/40) | 90  (36/40) | 15  (6/40) |
| 2007-08 | 48  (24/50) | 68  (34/50) | 86  (42/49) | 53  (26/49) | 33  (16/49) | 69  (34/49) | 100  (49/49) | 0  (0/49) | 98  (48/49) | 2  (1/49) | 94  (46/49) | 12  (6/49) |
| 2010 | 76  (13/17) | 59  (10/17) | 85  (17/20) | 15  (3/20) | 50  (10/20) | 50  (10/20) | 95  (19/20) | 5  (1/20) | 95  (19/20) | 5  (1/20) | 90  (18/20) | 15  (3/20) |
| **Sunyani** |  |  |  |  |  |  |  |  |  |  |  |  |
| 2003-04 | 35  (26/74) | 66  (49/74) | 57  (42/74 | 57  (42/74) | - | - | - | - | - | - | - | - |
| 2005-06 | 17  (6/35) | 86  (30/35) | 89  (31/35) | 31  (11/35) | 51  (18/35) | 49  (17/35) | 97  (34/35) | 3  (1/35) | 97  (34/35) | 3  (1/35) | 86  (30/35) | 14  (5/35) |
| 2007-08 | 58  (29/50) | 50  (25/50) | 96  (48/50) | 46  (23/50) | 52  (26/50) | 48  (24/50) | 98  (49/50) | 2  (1/50) | 100  (50/50) | 2  (1/50) | 90  (45/50) | 14  (7/50) |
| 2010 | - | - | - | - | - | - | - | - | - | - | - | - |
| **Tarkwa** |  |  |  |  |  |  |  |  |  |  |  |  |
| 2003-04 | 4  (2/50) | 98  (49/50) | 34  (17/50) | 96  (48/50) | - | - | - | - | - | - | - | - |
| 2005-06 | - | - | - | - | - | - | - | - | - | - | - | - |
| 2007-08 | 6  (1/18) | 94  (17/18) | 78  (14/18) | 67  (12/18) | 33  (6/18) | 67  (12/18) | 100  (18/18) | 0  (0/18) | 100  (18/18) | 0  (0/18) | 100  (18/18) | 11  (2/18) |
| 2010 | - | - | - | - | - | - | - | - | - | - | - | - |
| **Wa** |  |  |  |  |  |  |  |  |  |  |  |  |
| 2003-04 | - | - | - | - | - | - | - | - | - | - | - | - |
| 2005-06 | 23  (9/39) | 92  (36/39) | 74  (29/39) | 41  (16/39) | 56  (22/39) | 46  (18/39) | 92  (36/39) | 8  (3/39) | 97  (38/39) | 5  (2/39) | 87  (34/39) | 15  (6/39) |
| 2007-08 | 62  (26/42) | 95  (40/42) | 100  (40/40) | 38  (15/40) | 53  (21/40) | 48  (19/40) | 100  (40/40) | 0  (0/40) | 100  (40/40) | 5  (2/40) | 88  (35/40) | 12  (5/40) |
| 2010 | 70  (14/20) | 50  (10/20) | 85  (17/20) | 25  (5/20) | 45  (9/20) | 55  (11/20) | 100  (20/20) | 35  (7/20) | 100  (20/20) | 5  (1/20) | 95  (19/20) | 5  (1/20) |
| **Yendi** |  |  |  |  |  |  |  |  |  |  |  |  |
| 2003-04 | 52  (46/88) | 50  (44/88) | 67  (59/88) | 48  (42/88) | - | - | - | - | - | - | - | - |
| 2005-06 | 28  (11/40) | 73  (29/40) | 70  (28/40) | 40  (16/40) | 45  (18/40) | 55  (22/40) | 100  (40/40) | 0  (0/40) | 100  (40/40) | 3  (1/40) | 93  (37/40) | 13  (5/40) |
| 2007-08 | 42  (21/50) | 60  (30/50) | 100  (50/50) | 36  (18/50) | 40  (20/50) | 60  (30/50) | 98  (49/50) | 2  (1/50) | 100  (50/50) | 16  (8/50) | 94  (47/50) | 8  (4/50) |
| 2010 | 75  (15/20) | 45  (9/20) | 100  20/20 | 10  2/20 | 60  12/20 | 40  8/20 | 100  20/20 | 0  0/20 | 65  13/20 | 35  7/20 | 85  17/20 | 15  3/20 |

(-) data not available; figures are in percentages
